# International Society for Extracellular Vesicles: Second Annual Meeting, 17–20 April 2013, Boston, MA (ISEV 2013)

**DOI:** 10.3402/jev.v2i0.23070

**Published:** 2013-12-04

**Authors:** Elena Aikawa, Chris Gardiner, Joshua D. Hutcheson, Takahiro Ochiya, Xabier Osteikoetxea, Michiel Pegtel, Melissa Piper, Peter Quesenberry, Raymond M. Schiffelers, Tamás G. Szabó, Edit I. Buzas

**Affiliations:** 1 Cardiovascular Medicine, Brigham and Women's Hospital, Harvard Medical School, Boston, MA 02115; 2 Nuffield Department of Obstetrics and Gynaecology, University of Oxford, Oxford, United Kingdom; 3 Division of Molecular and Cellular Medicine, National Cancer Center Research Institute, Tokyo, Japan; 4 Department of Genetics, Cell- and Immunobiology, Semmelweis University, Budapest, Hungary; 5 Department of Pathology, VU University Medical Center, Amsterdam, The Netherlands; 6 Division of Pulmonary, Allergy, Critical Care and Sleep Medicine, Davis Heart and Lung Research Institute, Columbus, OH, USA; 7 Division of Hematology/Oncology, Rhode Island Hospital/The Miriam Hospital, The Warren Alpert Medical School of Brown University, Providence, RI, USA; 8 Division of Laboratories & Pharmacy Laboratory of Clinical Chemistry & Haematology UMC Utrecht, The Netherlands

During the Boston Marathon on 15 April 2013, two bombs exploded killing 3 people and injuring 264 others. The bombs exploded just one and a half days before the scheduled beginning of the ISEV 2013 Meeting. The leading news in world press was all about the Boston explosions when over 700 scientists of the extracellular vesicle field from all around the world chose to go to the meeting. The ISEV 2013 meeting was held as planned – a quiet triumph of science over terrorism.

## I. Opening of the meeting

Noting sympathy with all of Boston over the tragic recent events, J. Lötvall, President of ISEV, F. Hochberg, Head of the Local Organising Committee, and P. Quesenberry, one of the Chief Editors of Journal of Extracellular Vesicles (JEV), opened the second annual ISEV meeting in Boston.

## II. Oral sessions of the meeting

### 1. Biomarkers: urinary tract

In the past few years, urinary extracellular vesicles (EVs) attracted substantial attention as non-invasive biomarkers. Beyond the proteomic composition, several authors in Boston also presented data on the RNA patterns and functionality of urinary EVs both in tumorous and non-tumorous conditions. I. Bijnsdorp and colleagues (VU University Medical Center, The Netherlands) identified specific integrins in exosomes of prostate cancer cell lines. She presented data that the exosomal integrins were active and functioning as they facilitated the migration and invasion capacity of non-cancerous prostate cells. A significantly higher expression of exosomal integrins in urinary exosomes was found in patients with metastatic early-stage prostate cancer compared to benign prostate hyperplasia or localised prostate cancer. The authors concluded that exosomal integrins may play a role in prostate cancer metastasis, and could serve as a basis for risk stratification of prostate cancer metastasis. Next, M. Jayachandran (Mayo Clinic, USA) discussed that lithogenic molecules, such as oxalate and urinary crystals, may induce renal cell activation that is reflected by the protein composition of urinary vesicles. This finding broadens the spectrum of diseases in which EVs may serve as biomarkers to assess disease activity. In the next presentation, G. Deep (University of Colorado Denver, USA) suggested a mechanism by which hypoxia may induce a malignant phenotype in prostate cancer. Exosomes secreted by a prostate cancer cell line under hypoxia (1% O_2_) or normoxia (20% O_2_) were compared, and data were presented that exosomes secreted during hypoxia were loaded with unique signalling molecules and miRNAs that may confer enhanced invasiveness to prostate cancer cells. Focusing on another aspect of the question, C. Belleannée (Centre de Recherche du CHUQ/Université Laval, Canada) presented data that may help to fill the unmet need for non-invasive biomarkers to diagnose impaired sperm maturation. Seminal plasma EV miRNA signatures from normospermic, vasectomised and vasovasostomised donors were determined by microarray, and compared to arrays with miRNA signature from human epididymal tissues. The authors concluded that a specific subset of seminal plasma EV-miRNAs was derived from the epididymis, and may be used as non-invasive biomarkers to diagnose male infertility cases related to impaired sperm maturation.

### 2. EV biogenesis

More than 200 participants attended the session on biogenesis of EVs. First M. Colombo (Institut Curie, France) discussed results of an RNA interference screen targeting individual components of the ESCRT machinery in HeLa-CTIIA cells. She suggested a role of selected ESCRT components in exosome secretion and composition by HeLa-CIITA cells, and a role for ALIX in coordinating MHC Class II trafficking. She also provided evidence for biogenetic differences in vesicles secreted by different cell types. A presentation by H. Tahara followed (Hiroshima University, Japan) who spoke about the secretory mechanisms and functions of senescence-associated exosomes. He noted that there is a high production of exosomes in cellular senescence, and knock-down of maspin by siRNA inhibits exosome production in pre-senescent cells. Over-expression of maspin or CHMP4C increases the number of exosomes by three-fold. P. Zimmermann (Inserm-CRCM/K.U., France) described syntenin as a rate-limiting factor for the recycling and exosomal secretion of its cargo. She presented work on the downstream effectors and upstream regulators of “syntenin exosomes” showing that a small GTPase, ARF6, as well as a lipid-modifying enzyme, are involved in the formation of intraluminal vesicles within multivesicular endosomes. She mentioned that syntenin-ARF6 is at the intersection of endocytic recycling and the exosomal pathway. M van Hoek (George Mason University, Fairfax, VA, USA) discussed the role of increased membrane instability in higher outer membrane vesicle production in Francisella tularensis. Among the factors that increase membrane instability were mutations in the TOL/PAL system which also caused increased biofilm formation. She described the use of the outer membrane vesicles from Francisella tularensis as a novel vaccine candidate, based on positive results obtained with intranasal vaccination of mice. Finally, A. Wehman (Rudolf-Virchow-Zentrum, Germany) described the link between the lipid flippase TAT-5 and EV budding. Large scale shedding of EVs was observed with the loss of TAT-5 in *C. elegans* suggesting that the maintenance of lipid asymmetry by flippases is important for the regulation of EV budding. These results also suggest some shared mechanisms with viral budding.

### 3. Parasites and fungi

It is well known that vesicles are secreted by a wide variety of non-mammalian eukaryotes including uni- and multi-cellular organisms, as well as both dedicated and opportunistic pathogens. The first speaker of the session was A.C. Torrecilhas (Universidad Federal de Sao Paulo, Brazil) who presented work demonstrating a TLR2-dependent immunomodulatory role of vesicles secreted by *Trypanosoma cruzi*, which greatly enhances parasite invasion of host cells both *in vivo* and *in vitro*. Changes to the proteome of Leishmania infantum chagasi EVs released during different lifecycle stages was presented by B.K. Singh (University of Iowa, USA). M. Rodrigues (Universidad Federal de Rio de Janeiro, Brazil) gave an overview of EVs released by pathogenic fungi stressing that the wide diversity of vesicles secreted is likely a result of multiple mechanisms of cellular biogenesis. Proteins functioning at different steps along the secretion pathway, for example GRASP, SnF7p and Flippases, are all involved to some degree in EV production. However, EVs are still released by fungi when these proteins are rendered dysfunctional by mutation, leading Rodrigues to suggest vesiculation at the plasma membrane as at least one alternative mechanism of vesicle biogenesis. In support of this, using electron tomography of sequential sections he showed that fungal plasma membrane reshaping forms and releases vesicles directly into the extracellular space. Following this discussion of EV biogenesis, L. Nimrichter (Universidad Federal de Rio de Janeiro, Brazil) showed that *Candida albicans* EVs are internalised by dendritic cells and macrophages within 15 min. EV treatment stimulated cytokine production and modulated the antigen presenting phenotype of the cells. The EVs bound to the cell GM1 prior to internalisation, suggestive of a receptor–ligand interaction. Next, A. Buck (University of Edinburgh, UK) discussed the small RNA content of vesicles secreted by the gastrointestinal nematode *Heligmosomoides polygyrus*. Several hundred secreted pre-miRNAs were identified and stage specific secretion was observed. Notably, some of the highly abundant sequences were found to have the same seed sites as mouse miRNAs involved with regulation of immune responses. RNA-carrying EVs released by the parasite were absorbed by the small intestine epithelial cells of mice and worm-specific RNAs were internalised by epithelial cells during co-culture with vesicles *in vitro*.

In conclusion, EVs secreted by diverse pathogenic eukaryotes promote infection and invasion of host cells and tissues via multiple mechanisms, similar to findings with EVs from pathogenic prokaryotes. This suggests that employing EVs as a pre-emptive attack on target cells is an evolutionarily conserved pathogenic strategy.

### 4. Inflammation

The session covered a wide-range of topics from foreign body/tissue induced host responses, intercellular communication, and antigen presentation modalities. The session was opened by D.M. Pegtel (VU University Medical Center, The Netherlands) describing exosomal loading and transfer of the RNA polymerase III-transcribed small nuclear RNA EBER1 in inflammatory responses associated with the Epstein-Barr virus. Exosomal transfer of EBER1 (but not EBER2) is sufficient for inducing an interferon-mediated inflammatory response within dendritic cells. Further, exosomes with EBER1 were detected in sera of lupus patients, and EBER1 accumulation was observed in renal epithelial cells. Interestingly, these cells were found to be negative for Epstein-Barr virus DNA, suggesting that EBER1 is transferred through the host via exosomes in a manner that may be important for innate immune responses to the latent virus. The session continued with a talk by A. Morelli (University of Pittsburgh, USA) describing the role of exosomes in presenting donor MHC molecules to recipient dendritic cells following cardiac transplantation. The current paradigm of transplant recipient immune responses to donor tissues involves transplanted dendritic cells of donor origin that activate native responses. However, few donor dendritic cells are often found in these tissues – an observation that does not align with the large immune responses often seen in transplant patients. In the study presented, the authors demonstrated that exosomes containing donor MHC molecules are accepted in clusters by host recipient dendritic cells. Further, diptheria toxin depletion of host dendritic cells in CD11c-diptheria toxin receptor mice abolished T cell response to donor MHC molecules. These data indicate that paracrine signalling from native dendritic cells in the transplant recipient are necessary for the host immune response to the donor tissue, and may lead to a new paradigm of recipient–donor interactions in tissue transplantation. The session then moved to therapeutic usage of monocytic-derived exosomes loaded with the lipid α-galactosylceramide and ovalbumin in directing inflammatory responses against tumour growth. This study by S. Gabrielsson et al. from the Karolinska Institute, Sweden demonstrated that exosomal α-galactosylceramide and ovalbumin were more potent in inducing active immunity compared to soluble forms of these agents. Further, injection of exosomes containing α-galactosylceramide and ovalbumin into an ovalbumin-specific melanoma mouse model significantly increased survivability following tumour induction. A. Cumpelik (University Hospital Basel, Basel, Switzerland) followed with a presentation on the role of neutrophil-derived ectosomes in controlling inflammatory responses. The authors induced peritoneal inflammation by injection of mono-sodium urate crystals into the peritoneum of mice, and found a rapid release of the pro-inflammatory cytokine IL-1β. This response was quickly followed by the migration of neutrophils into the peritoneum that reached a maximum accumulation 8 h after the initial injection. Injecting the mice with neutrophil-derived ectosomes prior to the introduction of the mono-sodium urate crystals led to lower levels of IL-1β release and subsequent neutrophil migration. This response could be mimicked using phosphatidyl serine, but not phosphatidyl choline, liposomes. Further, endogenous neutrophil-ectosomes were found following neutrophil recruitment into the peritoneum. These results suggest that these ectosomes may be part of an auto-regulatory mechanism that is responsible for controlling the degree of inflammatory responses. The lecture in the Inflammation session given by D. Zecher (University Hospital Basel, Switzerland) focused on the role of microvesicles secreted by red blood cells during storage and potential transfusion-related problems with aged donor blood. The authors hypothesised that ectosomal shedding by donor red blood cells during storage induces systemic inflammation with the recipient. To test this hypothesis, ectosomes purified from aged mouse red blood cells were injected into recipient control mice of mice primed with LPS. No effect was observed in the control mice; however, mice primed with LPS and injected with the ectosomes exhibited significant pulmonary infiltration of neutrophils along with elevated levels of IL-6. These effects were inhibited in C5a receptor deficient mice, and the inflammatory responses in wild-type mice were mitigated by thrombin inhibition. Similarly to the findings in the previous lecture, the inflammatory responses studied were recapitulated by injection of phosphatidylserine liposomes. During the discussion portion of the lecture, the presenter mentioned that removal of ectosomes may be a strategy to prevent deleterious effects caused by aged donor blood. The final lecture in the Inflammation session delivered by P. Askenase (Yale University, USA) reported a novel means of miRNA transport whereby exosome-free miRNA associates with antigen-specific B cell exosomes to target effector T cells. This extracellular association confers antigen specificity to circulating miRNA, and these results explain observations that miRNA-150 induces immunosuppressive effects in an antigen-specific manner in a model of murine contact sensitivity. Traditionally, exosomal miRNA has been thought to exclusively originate from the exosomal source cell. Therefore, the results presented may represent a dramatic shift in our understanding of the interactions of miRNA and exosomes.

### 5. Detection technology

Standardisation of pre-analytical variables remains a major challenge in the analysis of EVs. R. Lacroix (Marseille, France) discussed recent progress upon the activity of the International Society on Thrombosis and Haemostasis (ISTH) Vascular Biology Standardization Subcommittee in reducing inter-laboratory variation due to sample preparation. Use of a common sample preparation protocol reduced inter-laboratory variability of flow cytometric enumeration of plasma EVs. J. Nolan (La Jolla, USA) stated that artefacts and irreproducibility represent the major obstacles to the use of EV measurement in routine clinical practice. The small size and low refractive index of EVs makes the analysis of small vesicles very difficult. However, this may be partially overcome by triggering on fluorescence rather than light scatter. Using a newly developed nanoparticle flow cytometer, optimised for analysis of individual membrane vesicles, it is possible to obtain a 5- to 10-fold improvement in fluorescence detection efficiency compared to a commercial instrument. This theme was continued by M.H.M. Wauben (Utrecht University, Netherlands), who reminded us that “EVs are no cells” and that their measurement requires a very different approach. Using a fluorescence triggering based high-resolution approach, it is possible to resolve nano-sized vesicles. However, it is important to titrate the sample to avoid simultaneous detection and measurement of multiple vesicles as a single event. This so-called “swarm detection” resulted in an underestimation of vesicle quantity and increased wide-angle forward scatter and fluorescence signal. H. Im (Massachusetts General Hospital, USA) described the application of plasmonic nanohole arrays which use surface plasmon resonance biosensors to study exosomes. Up to 12 antibodies, in duplicate, were captured via a PEG layer on a surface of a 24-hole nanoarray. The nanoholes displayed a high sensitivity for exosome detection and facilitated the identification of unique biomarkers for ovarian cancer detection. The performance of Nanoparticle Tracking Analysis (NTA) and Scanning Ion Occlusion Sensing (SIOS) for the characterisation of circulating EVs was discussed by S. Pedersen (Aalborg, Denmark). Both methods showed acceptable degrees of linearity and reproducibility. D. Kozak (Izon Science, USA) continued on the theme of SIOS (also known as tunable resistive pulse sensors [TRPS]), for size, concentration and charge measurements of nanoparticles.

### 6. Glioma-derived EV

Glioblastoma (GBM), which is the most common primary brain tumour derived from glial cells, is typically rapidly fatal. GBM cells and other types of cancer release EVs containing mRNA, microRNA, and proteins and can be used in diagnostic applications. In fact, B. Carter (University of California, USA) proposed the potential use of exosomal EGFRvIII RNA in plasma and CSF as a biomarker for GBM. M. Belting (Lund University, Sweden) demonstrated that GBM-derived EVs were rich in hypoxia-regulated mRNAs and proteins, representative of the producing cell, several of which were associated with poor prognosis. M. Sartori (University of Padova, Italy) showed that GFAP + /TF+ EVs were increased in GBM patients, particularly after tumour treatment, and that they correlated with disease progression. N. Atai (Harvard University/MGH, USA) showed that cellular uptake of exosomes was decreased in the presence of heparin, presumably because heparin mediated exosome aggregation. The analysis of exosomes and the determination of particle size, count, and components are crucially important. R. Weissleder (Massachusetts General Hospital, US) reported that a miniature nuclear magnetic resonance (µNMR) technique enabled differentiation of GBM-derived microvesicles from non-tumour host cell-derived microvesicles. M. Morello (Cedars-Sinai Medical Center, USA) presented his on-going study on the characterisation of GBM cell-derived large EVs, termed oncosomes. Electron microscopy demonstrated that some oncosomes contained smaller exosome-like particles in their luminal space, although their biological significance has not yet been characterised. These reports indicate that brain tumour EVs may provide more reliable therapeutic avenues to treat these malignant diseases.

### 7. Immune modulation

Earlier studies of S. Powis et al. (University of St Andrews, UK) identified the presence of dimeric MHC class I molecules on exosomes. In his presentation in Boston, he presented data on the ability of HLA-A and most HLA-B, but no HLA-C molecules to form disulphide-linked, dimeric structures on exosomes. G.E.R. Grau (The University of Sydney, Australia) discussed that human brain microvascular endothelial cell (HBEC)-derived microparticles expressed MHC I, MHC II, CD40 and ICOS. The vesicles enhanced T-cell activation suggesting a novel role for microparticles in neuro-immunological complications of infectious diseases. After this presentation, J. Dalli (Brigham and Women's Hospital and Harvard Medical School, USA) talked about lipid mediator metabololipidomics data, and showed that human neutrophil microparticles stimulate macrophage efferocytosis and contribute to macrophage biosynthesis of pro-resolving mediators during resolution of inflammation. The next speaker was Sadallah (University Hospital Basel, Switzerland) who showed earlier that platelet-derived microparticles (ectosomes) had suppressive activities on macrophages, DC and CD4 T cells. In the presentation at the Boston meeting, evidences have been summarised on the suppressive effects of platelet-derived vesicles on NK cell functions also. The last three presentations of the session included data obtained by gene expression microarrays. M.M. Keber (National Institute of Chemistry, Slovenia) has demonstrated that partially oxidised phospholipids in microvesicles from plasma of patients with RA or cells submitted to oxidative stress induce activation of TLR4 in cultured cells and *in vivo*. She discussed that hydro(pero)xyl phospholipids in microvesicles represent a ubiquitous endogenous danger signal released under the oxidative stress, which underlies the role of TLR4 signalling in inflammation. N.P. Bretz (German Cancer Research Center DKFZ, Germany) presented data on exosomes isolated from body fluids such as amniotic fluid, urine, liver cirrhosis ascites and malignant ascites from ovarian carcinoma patients. Irrespective of the source, exosomes triggered TLR-dependent signalling in THP-1 monocyte cells that was abolished by pre-treatment with proteinase K but not with DNase or RNase. The next speaker, T.G. Szabó (Semmelweis University, Hungary) provided evidence that soluble mediators (cytokines/chemokines) that are present simultaneously in the extracellular space along with EVs, have a cross-talk. Thus, the effects of soluble mediators in paracrine signalling may be substantially modulated by EVs. The last speaker of the session, L.A. Ortiz (EOH University of Pittsburgh, USA) presented data that mesenchymal stem cells transfer mitochondria and microRNAs by EVs as a mechanism for macrophage regulation. Macrophage uptake of exosomes reduced TLR expression and induced M1 polarisation.

### 8. Capture technology

Currently, the most widely used methods for EV isolation include differential centrifugation and sucrose gradient centrifugation. However, these methods are impaired by the possible overlap between different EV populations as well as protein complexes and other contaminants pelleting together. In this session, alternative capturing methods were presented which could circumvent some of the limitations of current isolation methods. S. Stott (Massachusetts General Hospital, USA) discussed the development and application of a high-throughput microfluidic chip designed for the isolation as well as characterisation of circulating tumour cells. The device was able to increase the amount of interactions with antibodies leading to an increased capture of these cells from blood. H. Shao (Massachusetts General Hospital, USA) presented a microfluidic device able to capture circulating glioblastoma multiforme microvesicles from volumes of blood smaller than 1 µL. The device was able to distinguish between gliobastoma and normal host cell derived microvesicles allowing for the identification of EGFR, EGFRvIII and PDPN as markers of glioblastoma derived microvesicles. The amount of these microvesicles could be correlated to tumour size. K van der Vos (Massachusetts General Hospital, USA) discussed a microfluidic device designed with 8 channels in a herring bone pattern and coated with antibodies against tumour antigens to mediate EV capture. EV RNA was isolated and analysed from the device. The following speaker, T. Ichiki (University of Tokyo, Japan) presented work on new methodology for single exosome analysis recording the exosomes’ zeta-potential shifts after reacting with anti-human CD63 antibody. The session continued with the presentation of D. Rupert (Chalmers University of Technology, Sweden) who discussed work on surface plasmon resonance detection of exosomes. With this method, low level of CD63 positive exosomes were detected upon binding to a biotinylated anti-CD63 antibody coated on a SPR gold chip. C. Helmbrecht (Particle Metrix, Germany) discussed the difficulty of characterising the zeta potential of exosome preparations with conventional methods due to their typically low concentration. However, with a different method, the zeta potential can be derived by measuring the electrokinetic migration of exosomes in a microelectrophoretic device. In addition, a laser and camera can be coupled to measure exosomes’ light scattering as they move in solution, allowing for the derivation of their size distribution using the Einstein-Smoluchowski relationship. Next, M. Rousseau (University of Laval, Canada) discussed the effect of secreted phospholipase A2 (sPLA2) on microvesicles. It was observed that sPLA2 could degrade microvesicles in biological fluids. Thus, it was recommended that studies in which microvesicle levels are measured upon different stimulations should also include measurements of sPLA2 levels in order to account for their ability to degrade microvesicles. Finally, S. Mathivanan (La Trobe University, Australia) discussed work comparing differential ultracentrifugation, epithelial cell adhesion molecule immunoaffinity pull down, and Opti-Prep™ density gradient. Isolation of exosomes from human blood plasma was performed with the three methods and revealed that Opti-Prep™ density gradient was relatively superior based on a lower amount of plasma proteins found in the isolations.

### 9. Platelet derived EVs

The first speaker of the session was E.B. Boilard (Université Laval, Canada). Using an autoimmune arthritis model and intravital imaging, he demonstrated that platelets may promote the accumulation of microvesicles outside the inflamed vasculature by stimulating the formation of endothelial gaps. This effect was mediated by platelet serotonin. This finding added to the rapidly growing list of unexpected functions for platelets. Next K. Witwer (Johns Hopkins University School of Medicine, USA) introduced ISEV Consensus on procedures for handling of biofluids for exosome analyses. He emphasised that as EV and exRNA research expands, important questions about technical protocols, standards for specimen handling and appropriate normative controls have been raised. In his talk, he outlined some of the considerations recently summarised by the first ISEV Position paper (Journal of Extracellular Vesicles 2013, 2: 20360). This presentation was followed by the lecture of B. Gyorgy (Semmelweis University, Hungary) who presented data on the prevention of artefactual *in vitro* platelet vesiculation in acid-citrate dextrose (ACD) anticoagulant tubes. He suggested the use of ACD tubes as anticoagulant tubes for subsequent flow cytometry analysis of circulating EVs to preserve blood sample vesicle concentration as close as possible to the *in vivo* conditions. Next, A. Piccin (San Maurizio Regional Hospital, Italy) presented data on microparticles and vascular markers in essential thrombocythaemia. The following speaker was B. Laffont (CHUQ Research Center/CHUL, Canada) who provided evidence for the presence of functional Ago2–microRNA complexes in platelet-derived microparticles that could exert extra-platelet mRNA regulatory effects in recipient HUVEC cells. In the following presentation, C.C. Chen (University of California San Diego, USA) discussed that the relative abundance of GADPH, 18S RNA, miR-103 and RNU49m in different cell line-derived EVs were low, and varied by an order of magnitude. Also, the authors found significant sample-to-sample variation in glioblastoma patients. Therefore they suggest that normalising EV content by the above genes may arbitrarily bias the quantitative analysis of miRNAs. L.H. Boudreau (Université Laval, Canada) showed that neutrophils release inflammatory lipid mediators upon incubation with platelet microparticle-associated immune complexes (generated *in vitro* by co-incubating microparticles and anti-fibrinogen) in an Fc receptor-dependent manner. The session was closed by the presentation of T. Wurdinger (VU University Medical Center, Netherlands) who presented data on the uptake of tumour-specific exosomes by platelets that contain mutated mRNA transcripts. He showed evidence that platelets are useful as a diagnostic platform for various types of cancer patients.

### 10. Plenary session

J. Quackenbush (Dana-Farber Cancer Institute, USA) gave a plenary lecture from the perspective of a computational biologist and genome scientist. He showed examples of alterations of protein–protein interactions based on microarray data, and emphasised applicability of Bayesian statistics.

The next plenary lecture was given by S. Gould (The Johns Hopkins University School of Medicine, USA). In his presentation, he discussed that the biogenesis of secreted vesicles can potentially occur at any organelle of the exocytic and endocytic pathways. Using a cargo-based approach, the relative contributions of these organelles to the budding of both acylated and integral membrane proteins were investigated. He presented data that indicate that the plasma membrane is the primary site of protein budding from animal cells, including human and fly cells.

Finally, A. Brisson (University of Bordeaux and IECB, France) presented data obtained using cryo-transmission electron microscopy (cryo-EM) imaging and receptor-specific gold labelling. With this approach, he reported the presence of three major types of EVs in normal human platelet-free blood plasma: he described smaller spherical objects (50–500 nm) representing 80% of the structures in blood plasma, tubular vesicles (15% of all MVs) and large objects (up to 7 µm). Strikingly, he found that only about 25% of vesicles bind Annexin V. He also found that about 20% of platelet-free plasma microparticles expose CD41, while most large cell fragments derive from red blood cells.

### 11. Prokaryote to eukaryote

D. Mulhall (University of British Columbia, Canada) gave a historical review of EV-related research, and encouraged the audience to utilise and consider the 50+ years of virus research as directly relatable to the investigation of EV biology. In addition to an overview of bacterial EVs, Y.S. Gho (POSTECH, Korea) presented EVpedia (www.evpedia.info), a high-throughput data collecting and mining site dedicated to EVs (1), which curates proteomic, lipidomic, and transcriptomic data from prokaryotes and eukaryotes, enabling interspecies comparisons. Finally, he discussed examples of the physiological relevance of bacterial EVs; for example the induction of septic shock by EVs alone (2), immune dysfunction in airways caused by EV treatment (3), and EV-mediated non-inherited β-lactam resistance in *Staphylococcus aureus* (4). T. Wei (Southwest Baptist University, USA) demonstrated that the production of EVs by *Pseudomonas aeruginosa* is sensitive to changes in the extracellular environment, including addition of antibiotics, and that vesicle release is increased under antibiotic-induced SOS conditions. Next, M. Sargiacomo (Instiuto Superiore di Sanita, Italy) demonstrated that EVs produced by mammalian cells that had been loaded with bacterial toxins (cholera toxin and cytotoxic necrotising factor 1) *in vitro* propagated those toxins from cell-to-cell, and did so in a more efficient manner than treatment with toxins alone. His data show that through EVs, a small population of cells can transfer and amplify a signal to a much larger group of neighbouring co-cultured cells, suggesting the technique could be broadly applied to investigation of EV-mediated cellular communication, endocrine function and homeostasis. The session was closed by S. Biller (Massachusetts Institute of Technology, USA) with a stimulating presentation on the release of EVs by the marine cyanobacterium *Prochlorococcus*. S. Biller showed that these organisms, which account for approximately 10% of global photosynthesis, release 70–100 nm vesicles – containing proteins, DNA and RNA – at concentrations approximately equal to the number of cells in the culture, 10^7^–10^9^ cells/mL. This was also true in ocean water samples; upper regions of the ocean had EV concentrations at 6 million vesicles/mL, similar to the number of bacteria, and this concentration was reduced in relation to depth. Moreover, EVs were found both in nutrient rich coastal waters and nutrient poor open ocean environments. S. Biller put forward three putative explanations for this massive vesiculation: (1) EVs are a vehicle for swapping of genetic material including the potential of cross species DNA sharing, (2) EVs provide defense against phages whereby the vesicles act as dummy cells, and (3) EVs deliver a source of fixed carbon to heterotrophic bacteria. S. Biller left the audience with the titillating estimation that potentially a full 10% of the earth's organic carbon is made up of extracellular vesicles in the oceans.

### 12. Heart and vessels

The lectures given in the Heart and Vessels session touched on a variety of cardiovascular issues such as angiogenesis, hyperlipidemia, calcification, and biomarkers for disease detection. The session was opened with a talk given by S. Sahoo (Northwestern University, USA) demonstrating hypoxia-induced changes in exosomal miRNA from CD34+ stem cells. A 2-dimensional electrophoresis analysis revealed few differences in protein expression between normoxic and hypoxic exosomes; however, miRNA analyses revealed that hypoxic exosomes were enriched in miRNAs-92a, 126, and 130a. Introduction of an anti-miRNA-126 construct led to reduced angiogenesis in an *in vitro* matrigel-based model as well as in an *in vivo* mouse model of hind limb ischemia, suggesting that miRNA-126 is necessary for angiogenesis and neo-vascularisation. The second presentation of the session, given by M.H. Nielsen (Aarhus University Hospital/Danish PhD School of Molecular Met., Denmark), identified a correlation between increased monocyte-derived microparticles in a 30-patient cohort of familial hypercholesterolemia. Along with the association with hyperlipidemia, the authors identified strong correlations between monocytic microparticles, CD16+ monocytes, and intima-media thickness. These monocyte-derived microparticles may be a measure of and participatory in inflammatory responses to elevated oxLDL in individuals with familial hypercholesterolemia. In the third lecture of the session, B.W.M. van Balkom (UMC Utrecht, The Netherlands) presented evidence that miRNA-214 is necessary for angiogenesis, endothelial cell migration, and vessel sprouting using both *in vitro* and *in vivo* models. Exosomal delivery of miRNA-214 to renal endothelial cells inhibited expression of the protein ataxia telangiectasia mutated, subsequently preventing senescence in recipient cells. Therefore, the data indicate that miRNA-2124 is crucial to the endothelial cell exosomal responses that lead to angiogenesis. The fourth lecture given by A. Kapustin (King's College London, UK) shifted the focus of the session to the biogenesis of smooth muscle cell-derived matrix vesicles that mediate vascular calcification. The data indicate that matrix vesicles arise from multivesicular bodies that form within vascular smooth muscle cells. Matrix vesicle release was elevated in synthetic non-contractile smooth muscle cells that arise from culture in calcific conditions, and shedding of matrix vesicles from these cells was abrogated by sphingomyelinase inhibition using SMPD3. Further, SMPD3 treatment suppressed calcification by smooth muscle cells *in vitro*. Finally, matrix vesicles were found to contain the exosomal markers CD63 and annexin A6, and both of these markers were observed in histological sections of calcified vascular tissue. Therefore, the data strongly indicate that calcifying matrix vesicles are exosomal in origin, and are produced by a phenotypic conversion of smooth muscle cells to a non-contractile phenotype. The fifth lecture of the session was delivered by D.P.W. de Kleijn (UMC Utrecht, The Netherlands Interuniversity Cardiology Institute of the Netherlands, The Netherlands, and National University & National University Hospital, Singapore). This study identified differential expression of four marker proteins in plasma microvesicles from patients that had a secondary cardiovascular event compared to patients that had experiences of only a single event. Of these markers, cystatin C, serpin F2, and CD14 MV levels were associated with increased hazard ratios for a secondary event. The results presented in this study point to an important set of biomarkers that may help identify those patients most at risk for secondary cardiovascular events. The final lecture in the heart and vessel session was given by G.E.R. Grau (The University of Sydney, Australia) and focused on the effects of monocytic microparticles on brain endothelial cell behaviour. In an *in vitro* model of sepsis, THP-1 monocytes were treated with LPS. This endotoxic activation led to enhanced annexin V positive microparticle (between 100 and 1,000 nm) release from the cells. Further, confocal microscopy indicated that these microparticles can be internalised by hCMEC/D3 brain endothelial cells in a manner that is blocked by inhibitors of endocytosis. Monocytic microparticles from LPS-activated THP-1 cells led to tighter endothelial cell junctions, modified Src tyrosine kinase phosphorylation, and enhanced endothelial cell microparticle production. These results indicate that microparticles from monocytes activated by endotoxin stimuli may play a role in modulating endothelial cell responses to sepsis infection.

### 13. Isolation technology

Reproducible isolation and purification of EVs from cell culture supernatants and body fluids remains a major challenge. C. Maguire (Massachusetts General Hospital, USA) described the use of heparin-agarose affinity chromatography to purify EVs from conditioned media from two cancer cell lines and HUVEC. The technique resulted in a comparable yield to ultracentrifugation and Exoquick™ with improved purity. Y. Yoshioka (National Cancer Center Research Institute, Japan) introduced ExoScreen amplified luminescent proximity homogeneous assay based on the AlphaLISA technique. This sensitive antibody capture based method enables the detection and quantitation of specific subsets of EVs having potential in cancer diagnostics. There have been many recent developments in microfluidic devices for EV isolation. N.V.J. Wise (University of Oxford, UK) explained the technique of “magnetophoresis,” which utilises monoclonal antibodies conjugated to para-magnetic beads in a continuous microfluidic system, and discussed the features necessary for successful EV isolation. Another microfluidic system using antibody-coated microbead capture of EV was described by J. Rho (Massachusetts General Hospital, USA). In this method, the isolated EVs were labelled with target-specific magnetic microparticles, which were then detected by miniaturised nuclear magnetic resonance. This facilitated antigenic profiling of EVs shed from stored erythrocytes. Field flow fractionation may be used to separate particles of different size, and this technique has been applied to the study of EV released from cancer cell lines by K. Agarwal (The Ohio State University, USA). Further analysis of miRNA showed that only a small number of secreted EV contained miRNA. E. Zeringer (Life Technologies, USA) reported on the latest commercially available techniques for exosome isolation and analysis.

### 14. Stem cells

Mesenchymal stem cells offer great potential in therapy for a wide range of conditions. Numerous data suggest that EVs are key players in mediating the effects of mesenchymal stem cells. In this session, the presentations highlighted novel roles of EVs vesicles in the context of stem cells. The session started with the lecture of S. Bruno (University of Torino, Italy) who discussed work showing that EVs derived from human mesenchymal stromal cells inhibited *in vitro* tumour cell proliferation and survival as well as the *in vivo* growth of established tumours. This presentation was followed by the lecture of P. Quesenberry (Rhode Island Hospital/The Miriam Hospital, The Warren Alpert Medical School of Brown University, USA). He presented data on experiments where rat lung derived EVs were able to induce epigenetic changes in mouse bone marrow, as evidenced by the induction of mRNA expression of surfactants A-D, aquaporin-5 as well as clara-cell-specific protein. Next, J. Morhayim (Erasmus University Medical Center, The Netherlands) discussed work on the characterisation of osteoblast-derived EVs and their effect and their interactions with human umbilical cord blood CD34+ haematopoietic stem cells. Membrane and signalling proteins linked to cell communication were found in osteoblast-derived EVs and they were found to promote expansion of CD34-expressing cells when incubated with CD34+ UCB-HSCs. The session continued with the presentation of T. Lener (Paracelsus Medical University Salzburg, Austria) who described the enhanced lineage induction of bone-marrow-derived mesenchymal stem/progenitor cells (BM-MSPCs) by exosomes derived from BM-MSPCs or endothelial colony-forming cells (ECFCs). BM-MSPCs derived exosomes promoted osteogenic and adipogenic induction while ECFCs derived exosomes induced proliferation. Next, L. Braccioli (University Medical Center Utrecht, The Netherlands) presented work about the role of mesenchymal stem cell (MSC) derived vesicles in neuroregeneration. By developing a novel co-culture system that allows for the exchange of soluble factors and exosomes, between MSC and neural stem cells (NSC), data were obtained showing that MSC vesicles could mediate the differentiation of NSC towards the neuronal and oligodendrocytic lineages. In the following presentation, S. Kiang (A*STAR Institute of Medical Biology, Singapore) described the role of mesenchymal stem cell (MSC) derived exosomes in mediating the efficacy of MSCs against immune diseases. Her work showed that MSC-derived exosomes have hypo-immunogenic capacity. These exosomes activated monocytes and enhanced the production of regulatory T-cell (Treg). C. Gardiner (University of Oxford, UK) described work on the use of EVs to assess the quality of IVF embryos in an attempt to determine those which are most likely to form pregnancies after transfer to the mother. It was observed that increasing EV size was strongly associated with decreasing embryo quality and that, with further studies, EVs may become a new parameter to determine IVF embryo quality. Finally, S. Weilner (University of Natural Resources and Life Sciences, Austria) discussed the effect of EVs released by senescent endothelial cells on mesenchymal stem cells. These EVs contained high levels of miR-31 and reduced the osteogenic differentiation potential of mesenchymal cells suggesting a possible role as a diagnostic and therapeutic target.

### 15. Virus and prions

K.M. Pate (Johns Hopkins University School of Medicine, USA) examined platelet activation and platelet microparticle formation during acute infection in the SIV-infected macaque model of HIV infection. She found platelet activation during acute SIV infection accompanied by increased platelet microparticle formation in some macaques. The next speaker of the session was T.C. Chen (Stanford University, USA) who discussed regulation of HCV RNA and MIR-122 by RAB27A-dependent exosome secretion pathway and showed that viral replication complexes were excluded from exosomes. P. Leblanc (CNRS, France) investigated the role of the components of the ESCRT machinery and the sphingomyelinase2-dependent ceramide biosynthesis in the exosomal release of infectious prions. The presented data suggested that prion accumulation within the cells can be differentially affected by the ESCRT machinery, and some ESCRT components and the ceramide pathway can also selectively inhibit exosomal release of prions. After this, A. Narayanan (George Mason University, USA) demonstrated the presence of TAR RNA in exosomes from cell culture supernatants of HIV-1-infected cells. The exosomes of HIV-infected cells contained host miRNA machinery proteins, Dicer and Drosha and distinct cytokines. Prior exposure of naïve cells to exosomes from infected cells increased susceptibility of the recipient cells to HIV-1 infection. Next, Gy Szabo (University of Massachusetts Medical School, USA) presented abundant data showing that exosomes in hepatitis C virus infection mediate CD81-independent transmission and are rich in Ago2-miR122-HSP90 complexes. Inhibiting miR-122 or HSP-90 inhibitors were shown to suppress exosomal transmission of HCV infection, suggesting their potential use in anti-HCV immune therapy resistant cases. The session continued with the presentation of A. Hubert (Université Laval, Canada) (HIV-1) who provided evidence for elevated amounts of exosomes in antiretroviral-naïve HIV-1-infected patients. These results indicate the involvement of microvesicles in mediating cell death and in the inflammatory response in HIV-1-infected subjects. The next speaker of the session, A. Kotani (Tokai University, Japan) provided evidences that EBV might utilise the exosomal machinery to secrete key viral-encoded miRNAs, through which EBV+ cells could modulate the tumour microenvironment. Finally, M.J. Kuehn (Duke University Medical Center, USA) referred to previous work in their laboratory that has shown that OMVs can bind the bacteriophage T4 irreversibly reducing the infectivity of the T4. T4-OMV complexes were found to represent a novel route of prophage induction across bacterial species.

### 16. Proteomics

C. Jimenez  (VU University Medical Center, The Netherlands) started the session discussing mass-spectrometry data obtained analysing 9 human cancer cell lines and 2 primary human cells comparing exosomes and the soluble secretome. The exosome-enriched proteins were associated with the ontology terms RNA post-transcriptional modification, protein synthesis and cell signalling, tumour-type-specific antigens and proteins belonging to oncogenic pathways. The next speaker was C. Lässer (University of Gothenburg/Krefting Research Centre, Sweden) who talked about using exclusion proteomics and quantitative proteomics to identify immune-related proteins in nasal exosomes. Exosomes were isolated from pools of nasal fluid exosomes from healthy controls, chronic rhinosinusitis (CRS) patients, asthma and asthma/CRS. Molecules associated with asthma susceptibility, such as mucins, were observed to be associated with exosomes of the asthma groups. The immunoregulatory S100 proteins were lower in the asthma/CRS patients. The session continued with the lecture of S. Kreimer (Barnett Institute of Chemical and Biological Analysis, USA) who discussed the results of a pilot survey of proteomic and posttranslational modification profiles of extracellular microvesicles isolated from the media of cultured MCF-7 and red blood cells, and blood plasma. This was followed by the presentation of G.E. Reid (Michigan State University, USA) who described significant differences between the exosome lipid profiles and their respective parent colorectal cancer cells, particularly for alkyl ether-linked glycerophosphocholine, alkenyl ether-linked glycerophosphoethanolamine and sphingomyelin lipids. Next, F. Dervin (Conway Institute of Biomolecular & Biomedical Research, Ireland) described that the platelet releasate, known to play a role in atherosclerosis, comprises both soluble and exosomal contents. Dervin quantified a panel of released proteins, and for the first time, released miRNA, also.

D. Di Vizio (Cedars Sinai Medical Center, USA) presented data obtained by the comparative proteomic analysis of the shedded “large oncosomes” (1–10 µm) that can be induced by over-expression of oncoproteins. She demonstrated a >90% overlap between the proteomic composition of large oncosomes and smaller size EVs, however, found 79 proteins enriched in large oncosomes (e.g. fibronectin) while canonical exosome markers were enriched in small EVs in comparison with large oncosomes. The session continued with the presentation of D.S. Choi (Pohang University of Science and Technology, Republic of Korea) who introduced data obtained by label-free quantitative proteomic suggesting that cellular proteins are specifically sorted into EVs. Vesicular proteins are mainly derived from cytosol, cytoskeleton, endosome and plasma membrane rather than other cellular compartments such as nucleus and mitochondria. Endosomal proteins and well-known vesicular marker proteins including CD9, CD81 and 14-3-3 proteins were only enriched in EVs. The last lecture was given by A. Lorico (Roseman University of Health Sciences, USA) who performed prominin-1-based immunomagnetic selection in combination with filtration and ultracentrifugation to isolate melanoma and colon carcinoma exosomes. The study suggested that specific populations of cancer exosomes contain multiple determinants of the metastatic potential of the cells from which they are derived (including CD44, MAPK4K, GTP-binding proteins, ADAM10 and Annexin A2). The authors found that the exosomes showed a great enrichment in lyso-phosphatidylcholine, lyso-phosphatidyl-ethanolamine and sphingomyelin, and exposure of MSC to prominin-1-exosomes increased their invasiveness.

## Late breaking abstracts

Among the speakers of the late breaking sessions, A. Arakelyan (Kennedy Shriver National Institute of Child Health and Human Development, USA) reported on the flow cytometric analysis of individual microvesicles and virions, using antibody capture on magnetic particles and multicolour flow cytometry. This was used to characterise EVs contained in HIV viral preparations and showed differences in CD45, CD81 and CD63 expression between EVs and virions. A novel assay platform using a membrane capture technique was presented by M. Mitsuhashi (Hitachi Chemical Research Center, Inc., Irvine, USA). Following capture, EVs were lysed prior to poly(A) RNA isolation, cDNA synthesis and gene amplification. J. Smith (Nanosight, UK) then outlined improvements to Nanoparticle Tracking Analysis software, which will lead to improved accuracy of sizing and concentration measurements of EVs. J. Costa (Laboratory of Glycobiology, ITQB-UNL, Portugal) characterised glycoproteins and glycans from exosomes of ovarian carcinoma cells using lectin blotting, immunoblotting and mass spectrometry. Costa and co-workers identified sialoglycoproteins enriched in exosomes. Distinct N-glycan profiles were also found for exosomes that had higher levels of sialylated glycans whereas the microsomal fraction was enriched in high mannose glycans. M. Dams (University Clinic Cologne, Germany) presented exciting evidence for the existence of a tubular/vesicular network that might contribute to tumour cell communication with distant infiltrating immune cells to contribute to the proinflammatory Hodgkin lymphoma microenvironment. According to these data, EVs were released and guided by a network of tumour cell-derived protrusions/cytonemes and caused a polarisation of CD30L-positive recipient cells. Z. Wang (The University of Texas at Austin, USA) developed fabrication protocols for novel ciliated micropillars (the micropillars with porous silicon nanowires on the sidewalls) as a convenient microfluidic tool for simultaneously multi-scale filtration of biofluids to isolate exosomes from complex biological samples. The authors validated their microfluidic system using both liposomes and biological fluids. Also among the late breaking presentations, S. Montoro-Garcia (University of Birmingham, UK) presented data on circulating small-size microparticles as indicators of worsening status in patients with systolic heart failure. K.R. Qazi (Karolinska Institutet, Sweden) investigated the mechanism of action of exosomes in sarcoidosis. More than 1500 distinct proteins were identified on the BALF exosomes. The expression of all the complement components was upregulated in the exosomes from patients. Conversely, CD55 and CD59 levels were higher in the exosomes from healthy controls. J.N. Leonard (Northwestern University, USA) has developed a system for engineering exosomal membrane proteins that bind to defined “packaging” sequences within engineered RNA cargo molecules. Using this engineered packaging system, the authors could direct the incorporation of specific proteins and RNA into exosomes. According to their data, there are novel active sorting mechanisms and/or biophysical constraints that dramatically modulate RNA loading into exosomes.

### 17. Plenary session

R. Langer (Massachusetts Institute of Technology, USA) opened the plenary session by focusing on the design of systems for drug delivery as a template for exosome studies. He emphasised that the critical problem for gene therapy has been delivery. He quoted I. Verma as once saying “the problem in gene therapy is delivery, delivery, delivery.” R. Langer first discussed the barriers to effective delivery and the methods that have been developed so far to address this critical problem. He noted that there are three types of delivery: systemic, local, and targeted. In the first two areas, he said there has been tremendous progress, but in the third it is still very early. He noted that two approaches (PLGA microspheres and PEGylation) have proven very effective and are now widely used in systemic delivery. In local delivery, stents, gliadel wafers (for the brain), and ureter tubes have been able to deliver high concentrations of drug to specific areas. With respect to specific traditional barriers, he mentioned that the transdermal barrier has been successfully hurdled by such approaches as the nitroglycerin patch. In regard to the lung, less than 2% of administered drug, such as from nebulisers, typically reaches the organ. However, recent studies have shown that the use of large porous aerosols actually changes everything and succeeds in delivering significantly more drug to the lung. R. Langer also discussed approaches to overcoming the barriers to oral delivery, mucosal delivery, and trans-vaginal delivery. In closing, he discussed systemic RNAi delivery via liposomes and noted that this approach is most effective in organs that have a fenestrated epithelium (e.g. the liver, spleen, bone marrow, and kidney). R. Langer's lab has developed a high-throughput combinatorial synthesis approach to produce thousands of different lipids to use in liposomes. R. Langer noted that there have been “remarkable potency improvements with novel lipid and lipid-like materials over the past eight years.” All of this work can provide useful paradigms for the study of exosomes for drug delivery.

### 18. Plenary Session

R. Langer was followed by C D'Souza-Schorey (University of Notre Dame, USA) who described the biogenesis of tumour-derived microvesicles. She noted that ARF6 is known to play multiple roles at the cell periphery and in her work she has shown a direct correlation between ARF6 activation and tumour progression. She noted that ARF6 activation promotes the formation of invadopodia which enhance tumour invasiveness, and also stimulates the biogenesis of microvesicles that are enriched for the active form of ARF6. She noted that there is selective sorting of cargo into the microvesicles and VAMP-3 is required for cargo delivery to sites. Inhibition of RAC-1 blocks the production of invadopodia and massively promotes microvesicle shedding.

D. Lyden (Cornell University, USA) closed the session by reporting on how tumour-derived exosomes promote pre-metastatic niche formation and organotropism. He emphasised that metastasis is an evolution and is not a late process; rather, it starts being developed early in the tumorigenic process. He said that secreted factors begin laying the foundation of the pre-metastatic niche at sites far removed from the primary tumour site very early. These secreted factors include growth factors, chemokines, hormones, extracellular matrix, microparticles and exosomes, and cell-free nucleic acids. He said that the tumour seems “to remember that I was once an embryo,” and seems to carry out reprogramming based on embryonic memory. He noted that tumour-derived exosomes increase lung endothelial permeability, causing vascular leakiness at this site. He added that melanoma-derived exosomes induce fibronectin formation in pre-metastatic niches. He reported examples of metastatic organotropism with melanoma specifically targeting the lung and liver and pancreatic cancer targeting the liver. In the question period, Dr. Lyden said it might be possible to re-direct tumour-derived exosomes to areas of the body that are more accessible to surgery.

### 21. Nanoparticles

In his talk, R.M. Schiffelers (University Medical Center Utrecht, The Netherlands) compared synthetic drug delivery systems with EVs. He overviewed several aspects including generation and loading of EVs with the desired compounds, shelf-life and colloidal stability, tissue targeting, target cell interaction, delivery of cargo and read-out of therapeutic effects. Next, P. Vader (University of Oxford) introduced a piece of work in which exosomes were isolated from HEK293T cells. Over-expression of shLuc or let-7b in HEK293T cells led to increased secretion via exosomes that were able to deliver the small RNAs to tumour cells *in vitro. In vivo* biodistribution in tumour-bearing mice showed accumulation of exosomes in liver, spleen and tumour tissue. The following speaker was D. Duelli (Rosalind Franklin University, USA) who provided evidence that malignant transformation induced *de novo* EVs, into which ex-miRs were assorted mutually exclusively. Uptake of ex-miRs and its consequences were cell-type and carrier-type specific. The presented data may explain how ex-miRs can simultaneously activate cancer-promoting cells and block anti-cancer cells. Later during the session, D.Y. Jeong (POSTECH, Republic of Korea) described the fabrication of exosome-mimetic nanovesicles by extruding living cells through constrictive microchannels. These nanovesicles were suggested to be used to study uptake and cellular material delivery pathway by EVs. This presentation was Y. Shu (University of Kentucky, USA) who reported on the fabrication of RNA nanoparticles with solid shapes, resistant to RNase degradation. Importantly, upon systemic injection, these RNA nanoparticles targeted cancer exclusively *in vivo* without trapping in normal organs and tissues. The presentation of K. Raemdonck (Ghent University, Belgium) pointed out some of the most unexpected findings of the meeting. The authors showed electroporation of induced aggregation of siRNA leading to overestimation of the efficacy of siRNA loading into EVs. The authors electroporated Cy5-labelled siRNA. Nanoparticle-tracking analysis (NTA) and confocal microscopy verified the emergence of large siRNA aggregates, likely due to the electroporation-induced release of aluminium cations from the cuvette electrodes. Thus, electroporation-induced precipitation may strongly bias the assumed encapsulation of siRNA into EVs. This presentation was followed by the one of A. Sehgal (Alnylam Pharmaceuticals, USA) who have developed a non-invasive method for following tissue-specific gene silencing monitored in circulating RNA.

### 22. Biogenesis and targeting

The first speaker, H. Christianson (Lund University, Sweden) provided evidence that heparan sulphate proteoglycans (HSPGs) function as internalising receptors of cancer cell-derived exosomes, and the uptake was inhibited by exogenous heparin sulphate but not with chondroitin sulphate. The uptake was dependent on intact HS 2-O-sulphation as well as N-sulphation. The lipid raft associated protein caveolin-1 regulated the uptake of exosomes negatively. The authors showed that exosome uptake appears dependent on unperturbed ERK1/2-HSP27 signalling, which is negatively influenced by CAV1 during internalisation of exosomes. The second speaker of the session, J.S. Lee (KAIST, Republic of Korea) developed photoactive therapeutic exosomes produced by tumour cells treated with membrane fusogenic liposomes loaded with therapeutic agents (photosensitisers). C. Villarroya-Beltri (CNIC, Spain) identified short RNA sequences over-represented in miRNAs enriched in EVs. The session continued with the lecture of F. Verweij (VUmc Cancer Center Amsterdam, The Netherlands) who described that palmitoylation of the EBV oncoprotein LMP1 in exosomes may represent an interesting analogous pathway to virion formation (during which incorporation in the budding virion of certain viral proteins depend on palmitoylation). Next, F.A. Court (Catholic University of Chile, Chile) proposed that exosomes mediate macromolecular transfer between Schwann cell and neurons to promote axonal regeneration and to improve nerve repair after peripheral injury.

J.C. Gross (German Cancer Research Center, DKFZ, Germany) showed that purified exosomes carry active Wnt proteins on their surface and can induce Wnt signalling activity in target cells. In the last presentation of the session, L. Corrigan (University of Oxford, UK) discussed data obtained using genetic tools in Drosophila. An important physiological role was shown for exosomes in seminal fluid, exosomes in sperm signalling and reprogramming female behaviour after mating, and the presented work revealed the dynamics of exosome formation and secretion *in vivo*.

### 23. Multiomics

S. Montgomery (Stanford University, USA) launched the Friday afternoon session on multiomics with a discussion of the genetics of gene expression in exosomes. He described his group's study of a three-generation, 17-member family for which every family member's genome had been sequenced and exosomal miRNA had been obtained for each individual. mRNA and miRNA libraries were created from cells and exosomes from family members and sequenced using a HiSeq instrument. The research team found a high correlation of exosomal miRNAs among the individual family members, both in cells and in exosomes. The team also found a huge enrichment for certain miRNAs in the exosomes. Montgomery noted that cell-specific regulatory variation could influence miRNA levels within exosomes. A. Clayton (Cardiff University, UK) followed by describing the quantitative proteomic analysis of cancer exosomes using a novel modified aptamer-based array (SOMAscan™). SOMA stands for Slow Off-Rate Modified Aptamers. In using this approach to analyse the protein composition of exosomes versus cells, he found these compositions to be “remarkably dissimilar,” in some cases varying by 200- to 300-fold. M. Kamali-Moghaddam (Uppsala University, Sweden) discussed advanced tools for sensitive detection of microvesicles and exosomes as biomarkers. He described a proximity ligation assay (PLA) for the detection of proteins and specifically reported on the use of a 4PLA-based detection of prostasomes. He noted that prostasomes in blood plasma can reflect the aggressiveness of prostate cancer. He also presented data demonstrating the high sensitivity of a multiplex proximity extension assay (PEA) for characterising prostasome surface proteins. He asserted that PLA and PEA approaches are highly specific, highly sensitive, and allow for multiplexing without an increase in cross-reactivity. I. Kurochkin (A*STAR, Singapore) reported that exosomes secreted by human cells transport largely mRNA fragments that are enriched in the 3’-untranslated regions. He noted that exosomes contain a large number of mRNAs that can be translated in another cell. Generally, the exosomal mRNA is smaller than the cellular mRNA, often being less than 500 nucleotides in length. He showed data indicating that 68.5% of mRNA in exosomes is fragmented. He also said that the 3’ ends of transcripts contain elements regulating stability, localisation, and translation activity of RNA. J.M. Falcon-Perez (CIC bioGUNE, Spain) followed with a talk on the application of omics-technologies to the study of extracellular vesicles in order to identify candidate non-invasive biomarkers for liver injury. He noted this is particularly important because of the difficulty of obtaining liver biopsies. In EVs from hepatocytes, proteomics analysis revealed 550 proteins; transcriptomics revealed 1300 gene transcripts; and metabolomics showed over 400 metabolites. He concluded that EVs are a valuable resource for disease markers and that protein candidates in serum could be useful in evaluating liver injury. A. Vlassov (Life Technologies, USA) discussed RNA profiling of exosomes. He initially described the workflow he is currently using. This involves isolation of exosomes, analysis, purification of RNA and protein, and then analysis of RNA and protein. He noted that the RNA cargo is primarily short RNA of 20–200 nucleotides. The cargo also includes some full-length mRNA and ribosomal RNA, and little or no DNA. He emphasised that exosomes are not the only entities containing extracellular circulating RNA. A significant fraction of circulating miRNA is bound to proteins rather than encapsulated in exosomes, he said. On average, each exosome contains 1–10 RNA molecules, he noted, assuming an average length of 100 nucleotides. Vlassov works at Life Technologies and he said that the company is planning to develop a complete arsenal of tools for exosome and microvesicle research. G. Schmitz (University Hospital Regensburg, Germany) closed the session by reporting that the multi-omics characterisation of subsets of platelet-derived extracellular vesicles (PL-EVs) suggests their involvement in the pathogenesis of vascular and neurologic diseases. He noted that PL-EV shedding increases during platelet storage and platelet-derived EVs (PL-EVs) are enriched in caveolin-1, ApoA-I/F and clusterin, alpha-synuclein, and contain amyloid precursor protein beta.

### 24. Plenary session

P. Sharp (Massachusetts Institute of Technology, USA), co-winner of the 1993 Nobel Prize for Physiology or Medicine for the discovery of RNA splicing, delivered a lecture to a full house in this plenary session. He began by noting that there has been an explosion in RNA biology in the last 10 years, which has been fuelled in part by advances that permit the sequencing of RNA at very deep levels and by the increasing awareness of molecules we previously did not even know existed. He emphasised the importance of studying the kinetics of RNA delivery to cells: what is delivered, how fast, and what happens after delivery. He reported that we now know that virtually any gene can be silenced by the appropriate siRNA and that the problem now is to translate this knowledge into useful therapeutics. As also noted by previous speakers, he said the critical problem is effective delivery of the therapeutic molecules. He noted that viruses solved this problem long ago. He said that liponanoparticles containing siRNA have proven highly efficient for specific gene silencing in hepatocytes. He asked the question of how much siRNA is needed to achieve gene silencing and reported that studies of factor VII siRNA in a mouse model indicated that 1 ng of siRNA per 1 gram of tissue was necessary and that this corresponded to 100 molecules of siRNA per cell. This would suggest, he said, that a single exosome would have to carry 100 molecules of siRNA to achieve specific gene silencing. He next suggested that a new modular approach to therapeutic entities could speed the development of new drugs and avoid the time-consuming problem of always re-inventing the wheel. He gave the examples of nanoparticle-drug, antibody-drug, lipid nanoparticles-siRNA/miRNA, and conjugates with siRNA as useful modules. He also reported the recent discovery of circular RNA that can sponge up miRNAs and noted that a decrease in regulation by miRNA is frequently observed in cancer development. He described a dual reporter technology that can be used to determine if there is miRNA control in a cell and, if so, to what degree. In the question section at the end of his talk, Sharp stressed the importance of more kinetics and more quantitation in RNA and exosome studies, and he closed by saying that “RNA biology is getting really interesting,” and emphasising that “nothing beats a biological assay, when you are looking for a biological process.

### 25. Endocrine/exocrine

Starting the session, L.F. Lincz (Calvary Mater Newcastle Hospital, NSW, Australia) described that elevated plasma levels of the fatty acid transporter, CD36, (mainly on the surface of erythrocyte derived microparticles), constitute a better biomarker for type 2 diabetes mellitus than circulating CD36 protein concentration. The following speaker, E. Buzas (Semmelweis University, Hungary) summarised recent advances in our knowledge of the roles of EVs in the induction, maintenance and regulation of inflammation. She highlighted the significance of the crosstalk between EVs and soluble mediators (such as cytokines or chemokines) in the paracrine signalling of cells. Next, S. Rome (CarMeN Laboratory (INSERM1060/INRA1235), France) indicated that she isolated and characterised mouse Quadriceps-released exosomes under physiological conditions or during insulin-resistance, and revealed that during insulin-resistance, myotubes release exosome-like vesicles which participate in functional alterations of pancreatic beta cells. The next speaker, D. Burger (Kennedy Kidney Research Centre, Canada) utilised a conditionally immortalised human podocyte cell line (HPOD) as well as two mouse models of progressive diabetic kidney disease. As discussed, podocytes produce ectosomes which are released into urine and may be indicative of glomerular injury. In her presentation, M. Frank Bertoncelj (University Hospital Zurich, Switzerland) showed that extracellular Poly (I:C) (PIC) associates with monocyte-derived microvesicles (MVs) and is transferred to rheumatoid arthritis synovial fibroblasts via MVs. The MV-delivered PIC may preferentially activate certain cellular pathways resulting in its pro-survival effects. Finally B. Bussolati (University of Torino, Italy) whose presentation was originally scheduled to Oral Session 1: Biomarkers: Urinary Tract, discussed that markers of inflammatory cells of endothelial damage and of cell regeneration can be detected in the urinary microvesicles of transplanted patients.

### 26. RNA analysis

The session started with the presentation of J. Chung (Massachusetts General Hospital, United States) who developed a new lab-on-chip system that can selectively enrich tumour-borne MVs and analyse their genetic information. The new fluidic-based platform can perform target-specific MV capture, their RNA extraction, and on-chip RT-PCR, and may prove useful both in basic and clinical research. The following speaker, R. Crescitelli (University of Gothenburg, Sweden) determined RNA profiles in exosomes, microvesicles and apoptotic bodies isolated from the supernatant of three different cell lines (HMC-1 human mast cell line, TF-1 erythroleukemia cell line and BV2 mouse microglia cell line), using two different centrifugation-based protocols. She demonstrated that the sub-populations of EVs have very different RNA profiles and morphological characteristics. The session continued with the lecture of ENM Nolte-'t Hoen (Utrecht University, The Netherlands). She separated EV sub-populations released during DC-T cell interactions by buoyant velocity gradient ultracentrifugation. Besides EVs that reach their equilibrium density of 1.15 g/mL after 14 h of ultracentrifugation (“fast-floating EV”), a specific subset of RNA-containing EVs reached this equilibrium density only after 60 h of ultracentrifugation (“slow-floating EV”). The authors found that during DC-T cell interactions, at least two different EV sub-populations are released that differ in migration velocity in density gradients, RNA content and morphology. Next, H. Akiyama (Toray Industries, Inc., Japan) compared the miRNA expression profiles of RNAs extracted from: (a) EVs precipitated by ultra-centrifuging, (b) precipitation of ExoQuick, (c) whole serum by QIAGEN miRNeasy, and (d) whole serum by Toray 3D-GeneTM RNA extraction reagent from liquid sample kit, and compared the serum miRNA expression profiles detected by: (a) “TaqMan” Array MicroRNA Card, (b) QIAGEN miScript miRNA PCR Array, and (c) “3D-Gene” Human miRNA oligo chip. At the end of the session, D. Gupta (University of ALLAHABAD, India) presented a computational approach for detecting candidate motifs and clustering patterns in miRNAs derived from exosomes.

### 27. Tissue injury

There was an impressive range of topics covered in the session. Vesicles as non-invasive placental biopsies in pregnancy were described by I. Sargent (University of Oxford, UK) and studies in preeclampsia of vesicles binding antibodies to cholera toxin recognising GM1 gangliosides or Annexin V recognising phosphatidyl serine showed elevations of preeclampsia related proteins and might represent biomarkers for preeclampsia (as discussed by T.S. Sim (Institute of Medical Biology, Singapore)). A number of presentations addressed the question of vesicle related tissue injury and recovery. There was a 6–8-fold increase in plasma vesicle levels in sepsis associated kidney injury (V. Cantaluppi, University of Turin, Italy). EVs cultured with kidney cells resulted in cell death, and blood purification using citrate anticoagulants led to decreases in plasma vesicle and cell injury. Vesicles from mesenchymal stem cells promoted renal cell recovery after ischemic injury (RS Lindoso; Carlos Chagas Filho Biophysics Institute UFRJ, Brazil). miRNAs may be involved in this process. Vesicles from endothelial progenitors also accelerated glomerular healing in anti-Thy1.1 nephritis through inhibiting complement injury and triggering angiogenesis. Monocrotaline treatment of mice induces pulmonary hypertension and vesicles from the plasma or lungs of these mice could induce pulmonary hypertension in normal mice (J. Aliotta; Rhode Island Hospital, USA).

### 28. Cancer: resistance and metastasis

Drug resistance is the most important cause of cancer treatment failure for chemotherapy. EVs derived from tumours have been found to enhance the development of drug resistance. M. Bebawy (The University of Technology Sydney, Australia) reported a novel cancer multi-drug resistance (MDR) pathway mediated by EVs *in vitro* and *in vivo*. EVs isolated from MDR breast cancer cell lines contained P-glycoprotein (P-gp), which is a drug efflux transporter protein, and functionally transferred P-gp to drug-sensitive breast cancer cell lines. T. Patel (Mayo Clinic, USA) reported that hepatocellular cancer (HCC) increased the expression of long non-coding RNA (lncRNA)-ROR in both cells and EVs compared to non-malignant hepatocytes. They also demonstrated that lncRNA-ROR in exosomes could modulate chemosensivity in HCC. It is well known that metastasis is also one of the hallmarks of malignancy progression and the major cause of treatment failure in human cancer. J. Kim (Cedars-Sinai Medical Center, USA) discussed potential diagnostic marker proteins in tumour-derived EVs for gefitinib-resistant non-small cell lung cancers (NSCLCs). EVs derived from NSCLC increased cell proliferation and invasion activity in recipient cells. Tumour-derived EVs have also been implicated in facilitating not only the development of drug resistance but also tumour invasion and metastasis. Indeed, T. Arscott (NCI/NIH, USA) showed the influence of radiation on EV secretion and signalling in glioma cells. Interestingly, radiation increased EV abundance and EV composition including CFGF mRNA, miRNA, and IGFBP2 protein. In addition, take-up of EVs derived from irradiated cells was increased, and they enhanced the migration and invasion activity of recipient cells. Heparanase was found to regulate secretion and function of tumour cell-derived exosomes. Increased heparanase increased exosome secretion, changed protein cargo, and stimulated spreading of tumour cells on fibronectin and invasion of endothelial cells (presented by C. Thompson, University of Alabama, Birmingham, USA). V. Luga (University of Toronto, Canada) demonstrated that fibroblast-secreted EVs promoted human breast cancer cell protrusions, motility, and metastasis via Wnt-planar cell polarity signalling. During the poster session, T. Katsuda (NCRI, UK) reported that osteosarcoma cells with knockdown of neutral sphingomyelinase 2 had decreased metastatic ability. However, systemic administration of original cell-derived EVs restored the metastatic ability of the knockdown cells. These findings suggest that further characterisation of EVs derived from tumours will lead to a better understanding of the appearance of drug resistance and tumour metastatic properties, which may improve cancer treatment.

### 29. Therapy: targeting and loading

The session began with M. Zöller's (University Hospital of Surgery, Germany) discussion of cancer immunotherapy and exosomes. She was followed by C. Lai (Massachusetts General Hospital, USA) who described the non-invasive *in vivo* imaging, tissue redistribution, and clearance analysis of intravenously administered EVs. V. Combes then spoke on unique roles for cytoplasmic actin isoforms in mechanically regulating endothelial microparticle formation. Next, M. Pegtel (VUmc, The Netherlands) described how comprehensive deep sequencing analysis revealed non-random small RNA incorporation into tumour exosomes and discussed the biomarker potential of these results. S. Kooijmans (University Medical Center Utrecht, The Netherlands) closed the session by speaking on how the loading of siRNA into EVs is accompanied by extensive siRNA aggregate formation.

### 30. Neurodegeneration

Several talks highlighted the role of EVs in neurodegeneration. First, G van Niel (Institut Curie/CNRS, France) described the presence of multilayer structures on the surface of exosomes reminiscent of synthetic amyloid oligomers in cryo-EM images. Further, van Niel reported that formation of these structures correlates with the presence and the processing of amyloidogenic domains of PMEL. Next, L. Rajendran (University of Zurich, Switzerland) proposed that exosomes are a major way to shuttle cytosolic proteins and amyloids out of the cell. Release of β-amyloid (Ab) peptides on exosomes aids in the plaque formation. C. Verderio (CNR Institute of Neuroscience, Italy) reported high production microglia-derived MVs in Alzheimer disease patients, and that MVs enhance Ab1-42 toxicity, thus providing a new link between microgliosis and AD degeneration. The session continued with the presentation by A. Cooper (Garvan Institute of Medical Research, Australia) who discussed the role of exosomes in Parkinson's disease proposing that extracellular transmission of toxic alpha-synuclein aggregates between neurons may serve as a basis for the neurodegenerative progression of Parkinson's disease. Cooper and co-workers discovered a protein whose expression level can modulate the extent of externalised alpha-synuclein associated with exosomes, potentially by influencing exosome biogenesis. The next speaker, A. Hill (University of Melbourne, Australia) reported that infectious prions were associated with intact, morphologically distinct exosomes. Hill and his co-workers found indication that intact vesicles are required for the successful transfer of prion infection. The RNA content of vesicles also demonstrated alterations in the levels of specific miRNA profiles between control and infected cells. The last speaker of the session, J. Skog (Exosome Diagnostics Inc., USA) reported the use of a platform to reproducibly extract high-quality CSF microvesicle RNA for RNA profiling from frozen biobanked CSF. He found a general down-regulation of the miRNAs in the Alzheimer patients and dysregulation of several miRNAs suggesting that CSF microvesicle RNA may be useful for the diagnosis of Alzheimer's disease.

### 31. Cancer: cell–cell communication

Cell–cell communication is an important tool for organisms and can be regulated through direct cell-to-cell contact, transfer of secreted molecules, or transfer of EVs. Recently, many reports have shown that EVs can be transferred between cells and that the contents of EVs, including protein, DNA, mRNA, and miRNA, are functional in recipient cells. D. Holterman (Caris Life Sciences, USA) demonstrated that the amount of exosomal miRNA in plasma was generally 5-fold higher in cancer patients than in healthy donors. E.A. Chiocca (Brigham and Women's Hospital, Harvard Medical School, USA) showed that exosomal miR-1, which is expressed at low levels in glioma, could inhibit angiogenesis through the down regulation of ANXA2 expression in recipient cells. H. Peinado (Weill Cornell Medical College, USA) reported that EVs derived from melanomas promote the metastatic behaviour of primary tumours by permanently “educating” bone marrow progenitors through the receptor tyrosine kinase MET. Moreover, they also demonstrated that the amount of exosomal TYRP2, which is a melanoma-specific gene, was significantly increased in melanoma patients’ plasma. J. Kim (Cedars-Sinai Medical Center, USA) revealed that EVs derived from DIAPH3-knockdown prostate cancer cells promoted cell proliferation and invasive activity in recipient cells and suppressed the proliferation of human immune cells. Several presentations focused on oncogene transfer using EVs. R. Simpson (La Trobe University) reported that EVs from oncogenic H-Ras over-expressing canine kidney cells induced EMT and the release of factors linked to promoting the metastatic niche, including transcription/splicing factors, in recipient cells. T. Lee (Montreal Children's Hospital Research Institute, Canada) presented data showing that “oncosomes” containing both mutant H-Ras protein and DNA could mediate the transfer of this oncogene into susceptible normal fibroblast cells, leading to long-term transformation (at least 7 days). It was also noted that stromal cell-derived EVs could inhibit tumour growth and malignancies. S. Bruno (University of Torino, Italy) reported that EVs from human mesenchymal stromal cells could inhibit tumour growth and survival *in vitro* and *in vivo*. Collectively, these reports demonstrate that the tumour microenvironment regulates malignant properties by eliciting reversible changes in the phenotype of cancer cells via EVs.

### 32. Therapy-stem cells

The first lecture on therapeutic aspects of stem cell derived EVs focused on their role in graft-versus-host disease (GVHD). L. Kordelas and colleagues (University Hospital Essen, Germany) reported a case-study regarding a successful therapeutic intervention with mesenchymal stem cell derived exosomes in therapy-refractory acute GVHD. Bone marrow derived stem cells were obtained from four independent donors. Immunosuppressive effects of exosome preparations were compared *in vitro* and the one exerting the most prominent effect was selected for therapeutic administration. The patient, a 22-year old female with therapy-refractory cutaneous and intestinal GVHD Grade 4, tolerated subsequent doses well and her symptoms improved significantly. In this case, MSC derived exosomes appeared to be a safe and effective therapeutic option for therapy-refractory GVHD. Motivated by the potential in EVs for specific and effective drug delivery, S.C. Jang and colleagues (Pohang University of Science and Technology, Republic of Korea), have described a new approach to generate artificial vesicles that mimic the features of exosomes. These bio-inspired vesicles were generated by the extrusion of monocytic cells through polycarbonate membranes. Cargo was loaded inside the artificial vesicles during the extrusion process. Delivery of cargo loaded into artificial vesicles during the extrusion process, was demonstrated in various models, including mouse tumour models for testing chemotherapeutics. Artificial vesicles described in the lecture, were not only found to be efficient for targeted delivery, but their yield has also been shown to be orders of magnitude greater than possible yields for exosomes. The potential therapeutic application of EVs of a genetically modified cell line was demonstrated by Z. Cai and colleagues (Zhejiang University, China). In the myelin oligodendrocyte glycoprotein (MOG) peptide-induced murine experimental autoimmune encephalomyelitis (EAE) model, the exosomes of membrane-associated TGF-ß1 gene-modified dendritic cells (mTGF-ß1-EXO) were found to prevent both the development and the progression of the disease. In the presence of mTGF-ß1-EXO, the level of cytokines associated with Th1 and Th17 immune responses decreased, in contrast to enhanced IL-10 production and increased numbers of Treg cells. Furthermore, this immunosuppressive effect was not abrogated, even when mTGF-ß1-EXO of C57BL/6 mice were administered to another murine strain, Balb/c.

The factors secreted by stem cells seem to be at least as important as the cells themselves in therapy and one of the factors secreted by MSC is EVs. R.C. Lai and colleagues (Institute of Medical Biology, Singapore) were focusing on how these structures can exert their effect. Exosomal cargo was analysed by mass spectrometry in order to identify biochemical reactions potentially modulated by exosomal proteins. Key functions predicted to be affected, included glycolysis, activation of kinase pathways, reducing complement activation and increasing proteasome function. Modulation of the predicted biological functions was also confirmed by the appropriate enzymatic or cellular assays.

How cell differentiation and survival might also be affected by the presence of EVs *in vitro*, was demonstrated by R. Andriantsitohaina and colleagues (INSERM, France). Administering microparticles of activated or apoptotic lymphocytes to human umbilical vein endothelial cells resulted in a pro-angiogenic and anti-apoptotic effect. Scavenging reactive oxygen species and carrying antioxidant enzymes might be an important factor in the anti-apoptotic effects of EVs. Both pro-angiogenic and anti-apoptotic effects were abolished however, in the presence of a pharmacological inhibitor of Sonic Hedgehog signalling, cyclopamine. In contrast to the previous report on anti-apoptotic effects, V. Huber and colleagues (Fondazione IRCCS Istituto Nazionale dei Tumori, Italy) propose an anti-tumour strategy based on modified EVs inducing apoptosis. The apoptotic signal TRAIL was expressed by the K562 cell line in a membrane-bound form and also detected in exosomes secreted by these cells. The proapoptotic effect of TRAIL containing exosomes could be demonstrated *in vitro* on haematological cancer and melanoma cell lines, but only a moderate effect was observed *in vivo* on tumour progression.

### 33. Bacteria-infection

E. Ligeti (Semmelweis University, Hungary) launched the session on bacteria and infection by showing data demonstrating that neutrophil-derived anti-bacterial microvesicles are increased in bacteremic patients. Next, F. Vazirisani (University of Gothenburg, Sweden) discussed the effect of membrane vesicles from *Staphylococcus aureus* and *Staphylococcus epidermidis* on monocytes and macrophages. R. Ovstebo (Oslo University Hospital, Norway) then reported that the inhibition of complement protein 5 reduces *Neisseria meningitidis*-induced microparticle-associated tissue factor activity as measured by thromboelastography in whole blood. J. Schorey (University of Notre Dame, USA) described the characterisation of exosomal RNA from M. tuberculosis-infected macrophages and discussed the potential use of these RNAs as molecular biomarkers for tuberculosis. Finally, Y. Cheng (University of Notre Dame, USA) reported that exosomes containing mycobacterial antigens induce a protective immune response against M. tuberculosis infection in mice.

## III. Poster presentations

This report cannot undertake the task to individually discuss the 243 posters presented in Boston. Therefore, here we will refer to a limited number of important posters with special focus on those awarded as outstanding posters by the ISEV Award Board.

An interesting poster demonstrated that E. coli-derived vesicles were found to induce emphysema via both IFN-gamma and IL-17 dependent pathways and vesicles from activated fibroblasts enhance fibrosis in a Bleomycin lung injury model (J.-P. Cho, POSTECH, Republic of Korea).

In neural studies, neuronal exosomes were shown to eliminate Alzheimer's amyloid protein (K. Yuyama, Hokkaido University, Japan). These results suggest that amyloid-β protein binds and assembles on the surface of exosomes and is delivered into microglia to degrade together with exosomes. In a similar vein, exosomal mediated release of alpha-synuclein had implications for Parkinson's disease (G. Thakur, University of Zurich, Switzerland). Furthermore, endothelial ectosomes were found to be elevated in a rodent model of cerebral hypoperfusion (D. Burger, University of Ottawa, Canada). These particles cause apoptosis in cultured cells and a decrease in blood brain barrier permeability *in vivo*.

Endothelial cell-derived microparticles were also found to exert catabolic effects on intervertebral discs (P.H.I. Pohl, University of Pittsburgh Medical Center/The Ferguson Lab For Or, USA).

There were many posters on both the beneficial and the detrimental effects of vesicles on cancer. Breast tumour-derived vesicles enhanced EMMPRIN-dependent human breast cancer invasion (K. Menck, University Medical Center Gottingen, Germany). Breast and melanoma derived vesicles were found to directly influence metastatic sites in the lung and to modulate marrow cells to further influence metastases (T.-L. Shen, National Taiwan University, Taiwan). Exosomes from brain metastases breast cancer cell were also shown to promote the blood brain barrier destruction (N. Tominaga, National Cancer Center Research Institute, Japan).

There were also a number of posters related to prostate cancer. There was reversal of resistance of prostate cancer cells to campothecin and anchorage independent growth in soft agar by vesicles from a normal prostate cell line (K. Panagopoulos, Rhode Island Hospital, USA). Conversely, prostate cancer exosomes could promote prostate cancer progression via activation of ERK cell pathway (E.H. Beheshti, The Vancouver Prostate Centre (UBC), Canada). Of interest was a report showing that epithelial mesenchymal transition could be induced in normal prostate cell lines by MVs from a T cell line (Jurkat) (J.M. Inal, London Metropolitan University, UK).

Gist exosomes mediated transformation and had a role in tumour spread while microparticle-mediated transfer of ABC drug resistance was shown and an association of exosome secretion and drug resistance in aggressive B-cell lymphomas (S. Atay, University of Kansas Medical Center, USA) was demonstrated. Diminished exosome release was associated with increased efficacy of imunochemotherapy.

Melanoma cell derived exosomes re-educate mesenchymal stem cells through their miRNA cargo giving rise to a population that supports metastases (M. Harmati, Hungarian Academy of Sciences, Biological Research Centre, Hungary).

Regarding the biogenesis of EVs, V. Hyenne and colleagues (IGBMC, France) focused on the intracellular mechanisms involved in trafficking exosome-containing MVBs to the plasma membrane. Studying exosome biogenesis in *C. elegans*, the authors could exploit the advantages of studying a multicellular organism with live imaging techniques, as well as molecular genetics. Based on their previous observation that EVs contribute to alae formation (part of the cuticle), RNAi screening yielded over candidate 60 genes. Confirming subsequent changes in MVB morphology by EM, the exocyst protein complex and its regulator, ral-1 were found to be important in the mechanism of exosome release.

Innovative approaches for isolation and detection of EVs were presented by many posters at the meeting. Among these attractive posters, R. Baek's (Aalborg University Hospital, Denmark) approach has engaged an outstanding amount of interest. With his colleagues, he has utilised protein microarray technology to screen for 21 potential EV markers (including CD9, CD63 and CD81) in the blood of 80 healthy donors. When comparing the expression of these surface markers on EVs to the number of exosomes in the donors’ plasma, they found that EVs from individuals with a lower exosome count have a higher abundance of exosome markers on their surface.

EV secretion is an evolutionary conserved feature, as also demonstrated by many posters. Among data on EV secretion by bacterial cells, the poster proposing a new mechanism of antibiotics resistance conferred by EVs, attracted significant interest. J. Lee, with his colleagues (Pohang University of Science and Technology, Republic of Korea) studied the role of *Staphylococcus aureus*-derived EVs in inter-bacterial communication. By exposing ampicillin-sensitive bacteria to EVs of ampicillin-resistant bacteria, the authors found that the exposed bacteria became insusceptible to the effects ampicillin. Since the survival was transient and EVs did not contain the resistance gene blaZ, this effect was most likely mediated by the gene product itself, the beta-lactamase enzyme present in EVs. Furthermore, the release of beta-lactamase containing EVs by resistant bacteria was enhanced in the presence of ampicillin.

Beyond inter-bacterial communication, EVs might be important in intercellular communication of a multicellular organism as well, as demonstrated by A. Lo Cicero and colleagues (Institut Curie, France). Within the epidermal melanin unit, melanocytes supply keratinocytes with pigment melanin and keratinocytes provide feedback to melanocytes. It has been known that soluble mediators are involved in this feedback, but this work places EVs in the model also. The results showing that melanocytes isolated from Caucasian donors increase the level of melanin in the presence of exosomes derived from the keratinocytes of Black donors, clearly supports this concept.

Uncovering the RNA content of EVs promises therapeutic and diagnostic development, like RNA delivery by EVs. In order to monitor this process, we need to track both EVs and their cargo. C.P. Lai and his colleagues (Massachusetts General Hospital, USA) described a fluorescent reporter-based solution. They added a palmitoylation signal at the N-terminus of GFP or tdTomato and then tagged the transcripts with MS2 RNA-binding sequences. This way, the constructs were targeted to EV membranes by the palmitoylation signal, as well as capable of RNA binding. Whether RNA was actually bound to the tagged protein, was detected by bacteriophage MS2 coat protein fused to GFP. Using this approach, co-localisation of EVs and their RNA cargo could be demonstrated. Furthermore, simultaneous assessment of the fate of EVs and RNA after uptake by recipient cells also became possible.

M.G. Amorim and her colleagues (A.C. Camargo Hospital, Brazil) drew EV researchers’ attention to how the over-expression of one single oncogene may alter proteomic landscape of EVs. When comparing EVs of HB4a human mammary luminal epithelial cell line and its transformed clone over-expressing HER2, proteomic analysis showed notable differences in the presence and relative amount of proteins. Over-expression of HER2 resulted in both up- and down regulation of vesicular proteins. This observation is very important when considering using constructs over-expressing a reporter, since this may also affect the relative abundance of vesicular proteins.

Loading EVs with nucleic acids, however, is therapeutically even more promising than modifying the protein content. A. Banizs and colleagues (University of Virginia, USA) reported important results on the potential of endothelial cell derived EVs as nucleic acid vectors. The authors have provided comprehensive data on quality, morphology and biodistribution of mouse aortic endothelial exosomes. Additionally, it was also shown that endothelial exosomes are able to bind to endothelial cells and deliver functional siRNA into these cells, thus being capable of nucleic acid delivery both *in vitro* and *in vivo*.

Furthermore, in another model system, mesenchymal stem cell derived exosomes were found to be protective in hypoxia induced pulmonary arterial hypertension (PAH) by K. Sdrimas and colleagues (Children's Hospital, Boston, USA). Mesenchymal stem cell derived exosomes were found to prevent remodelling of vascular smooth muscle cells, triggered by hypoxia. The presence of exosomes inhibited the hypoxic activation of STAT3 and the increase in a-smooth muscle actin (a-SMA) mRNA and protein levels, as well as prevented the migration of lung fibroblasts and counterbalanced the effects of PDGF on vascular smooth muscle cells.

Another clinically relevant aspect of EV RNAs is their role as potential biomarkers of various diseases. K O'Brien and colleagues (Trinity College Dublin) have thought a step ahead, and investigated the clinical relevance of exosomal miRNAs in triple-negative breast cancer. Exosomes were isolated from Hs578T breast cancer cell line and its more invasive variant, Hs578Ts(i)8. Analysis of RNAs present in EVs of the two cell lines, suggests that tumour-related exosomal RNAs also have a functional role beyond being mere markers of the disease.

The role of EVs in promoting tumour progression is further supported by the results presented by T. Katsuda and colleagues (National Cancer Center Research Institute, Japan). Proposing a scenario, where osteosarcoma exosomes educate the lung microenvironment in order to enhance metastasis formation, the 143B human osteosarcoma cell line was modified to lose the ability of exosome secretion, by shRNA knockdown of neutral sphyngomyelinase 2. When these cells were transplanted to nude mice, tumour cells incapable of exosome secretion developed a lung metastasis with a lower frequency as compared to exosome secreting 143B cells. When 143B derived exosomes were also administered to mice transplanted with 143B cells devoid of exosome secretion capability, frequencies of lung metastasis were restored to the original level. These results support that osteosarcoma-derived exosomes enhance tumour progression by preparing a pre-metastatic niche.

Concerning that EVs may promote tumour progression, it is also interesting whether EV dynamics are altered during therapeutic intervention. This question was addressed by K.A. Wong and colleagues (Tufts Medical Center, USA), in a mouse model of glioblastoma multiforme, characterised by the over-expression of EGFR and impaired Cdkn2a function. In this system, EGFR inhibition therapy resulted in differential sorting of EV cargo, in parallel with an increased number of tumour infiltrating microglia. Given that microglia were shown to take up EVs derived from primary tumour cell cultures, data presented here suggests that cargo altered by the therapeutic intervention contributes to the formation of a different microenvironment.

M. Bürdek, with her colleagues (Fondazione IRCCS Istituto Nazionale Tumori, Italy) focused on the role of EVs in anti-tumour immunity. T cell reactivity to melanoma cells derived exosomes was assessed by IFN-γ ELISPOT, cytokine release assay and detection of activation markers (CD25 and CD137) by flow cytometry. HLA-A2, a key player in melanoma-specific immune reaction, was also detected on exosomes derived from HLA-A2 expressing cells and found to be important in eliciting CD8+ T cell response, as suggested by comparison of results with HLA-A2-restricted T cell clones and oligoclonal cell lines. Although the nature of the immune response against tumour derived EVs is still unclear, it is very important to see that they do elicit T cell responses.

Further assessing immunological features linked to EVs, M. Mossberg and colleagues (Lund University, Sweden) focused on the role of EV in vasculitis. The authors provided evidence on the presence of complement C3 and C9 on the surface of endothelial cell derived microparticles in patients with vasculitis. Microparticles were assessed by flow cytometry and endothelial origin was determined by CD105 and CD144 staining. Patients had significantly higher levels of endothelial microparticles as compared to healthy controls, as well as higher levels of both C9 and C3.

## IV. Closing ceremony

During the ISEV 2013 closing, J. Lötvall and the Awards Board of ISEV presented 14 young investigator awards for outstanding poster presentations and 9 young investigator awards for outstanding oral presentation. The Board made its selections from a total of 243 poster presentations and 184 oral presentations.

The winners for outstanding poster presentations were Vincent Hyenne, Jaewook Lee, Maria Amorim, Rikke Baek, Charles Ping-Kuang-Lai, Katy Wong, Konstantinos Sdrimas, Maria Mossberg, Maja Burdek, Kohei Yuyama, Alessandra Lo Cicero, Keith O'Brien, Anna Banizs, and Takeshi Katsuda.

The winners for outstanding oral presentations were Stephen Biller, Stefania Bruno, Carolina Villarroya-Beltri, Luis Ortiz, Thomas Wurdinger, Susmita Sahoo, Aled Clayton, Muthuvel Jayachandran, and Esther Nolte-'tHoen.

Next, a richly deserved ISEV Special Achievement Award was presented to President J. Lötvall.

This was followed by the noting of meeting highlights in the area of clinical applications as described by P. Quesenberry and general highlights as described by M. Wauben. Of the 427 abstracts, P. Quesenberry considered 101 as being of significant clinical interest. These included many in the areas of solid and haematological cancers. In addition, a significant number addressed neurologic diseases such as Alzheimer's and Parkinson's. Miscellaneous subjects of clinical interest included a study of smoking, exosomes, and lung cancer; and the use of photoactive vesicles for cancer therapy. P. Quesenberry predicted that clinical trials with microvesicles will be a critical feature of ISEV meetings within 2–3 years.

M. Wauben pointed out that many of the most important meeting highlights for her were comments that she heard from scientists as she moved around the meeting and attended the various poster sessions. The posters, in fact, are “in the heart of our society,” she said. She provided some data to document the progress of the society. The first international workshop in Paris in 2011 featured 115 abstracts and focused on exosomes. The first ISEV Congress in 2012 featured 255 abstracts and focused not only on exosomes, but also on microvesicles/microparticles. This year's ISEV Congress featured 427 abstracts and focused on exosomes, microvesicles, and outer membrane vesicles from bacteria. This progress came together with a significant increase in cross-talk between and among different disciplines. One strong message coming out of this year's meeting, M. Wauben said, is that microvesicles “offer a tremendous opportunity for clinical applications.”

Next, J. Lötvall announced that the site for the 2014 ISEV annual congress would be Rotterdam, The Netherlands in 2014 and scheduled to take place 30 April–3 May 2014.

Finally, J. Lötvall and F. Hochberg declared that the ISEV Boston 2013 meeting was finished.

